# Checklists in the operating room: Help or hurdle? A qualitative study on health workers' experiences

**DOI:** 10.1186/1472-6963-10-342

**Published:** 2010-12-20

**Authors:** Øyvind Thomassen, Guttorm Brattebø, Jon-Kenneth Heltne, Eirik Søfteland, Ansgar Espeland

**Affiliations:** 1 Department of Anaesthesia and Intensive Care, Haukeland University Hospital, Bergen, Norway; 2 Department of Research, Norwegian Air Ambulance Foundation, Drøbak, Norway; 3 Department of Medical Sciences, University of Bergen, Bergen, Norway; 4 Betanien University College, Bergen, Norway; 5 Department of Radiology, Haukeland University Hospital, Bergen, Norway; 6 Department of Surgical Sciences, University of Bergen, Bergen, Norway

## Abstract

**Background:**

Checklists have been used extensively as a cognitive aid in aviation; now, they are being introduced in many areas of medicine. Although few would dispute the positive effects of checklists, little is known about the process of introducing this tool into the health care environment. In 2008, a pre-induction checklist was implemented in our anaesthetic department; in this study, we explored the nurses' and physicians' acceptance and experiences with this checklist.

**Method:**

Focus group interviews were conducted with a purposeful sample of checklist users (nurses and physicians) from the Department of Anaesthesia and Intensive Care in a tertiary teaching hospital. The interviews were analysed qualitatively using systematic text condensation.

**Results:**

Users reported that checklist use could divert attention away from the patient and that it influenced workflow and doctor-nurse cooperation. They described senior consultants as both sceptical and supportive; a head physician with a positive attitude was considered crucial for successful implementation. The checklist improved confidence in unfamiliar contexts and was used in some situations for which it was not intended. It also revealed insufficient equipment standardisation.

**Conclusion:**

Our findings suggest several issues and actions that may be important to consider during checklist use and implementation.

## Background

The effectiveness of checklists has been demonstrated in various medical fields [1-4]. One example is WHO's Safe Surgery checklist, which has been implemented in over 3,000 hospitals worldwide [5]. Checklists can improve information exchange among operating room (OR) team members; however, the attitudes of personnel toward checklists may vary, and attitudes and experiences are likely to influence compliance [6-9].

Few groups have studied the experiences and acceptance of health workers regarding checklists [6,7]. It has been stated that checklists "might seem deceptively simple, but the effective use of them is a complex issue that encompasses different groups within the health care system and organisational change"[10]. Hence, to promote the success of checklists, we must clarify their acceptability and their effects on relationships among health professionals. Because little is known about these issues, a qualitative study could provide valuable insight [11].

**Figure 1:**
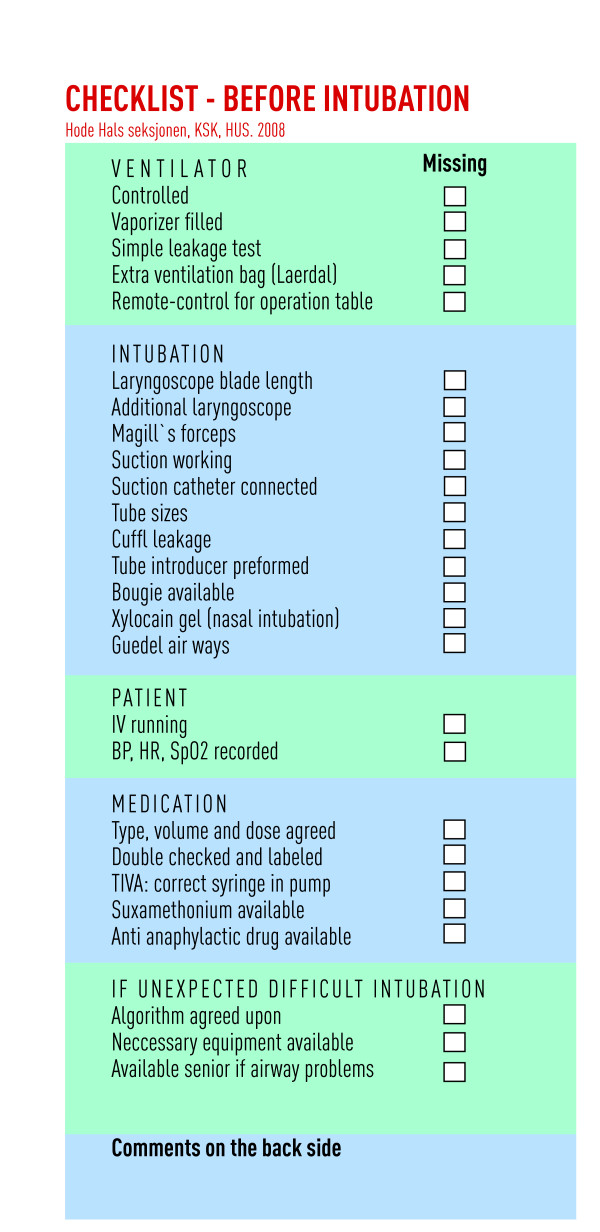
**The checklist that was implemented in our department in 2008**.

In 2008, a pre-induction checklist was introduced into one section of the Anaesthesia and Intensive Care Unit of a 1,100-bed tertiary teaching hospital [12]. The purpose of the checklist was a double check of equipment, patient preparation, medication, and the preparedness for an unexpected difficult intubation (Figure 1). As reported elsewhere, the checklist identified and reduced a large number of missing items[12]. The aim of the present qualitative study was to explore nurses' and physicians' acceptance and experiences with this checklist.

## Methods

This study was based on focus group interviews [13,14]. The study was approved by the Institutional Research Ethics Committee and the Norwegian Social Science Data Services. All study participants gave their informed consent to participate.

### Study setting

In 2008, 26 nurse anaesthetists, four consulting anaesthetists, and four residents that served seven operating theatres (neurosurgery, plastic surgery, burn surgery, and otolaryngological surgery) participated in developing, implementing, and using the pre-induction checklist. During the development process, the list was revised several times using a modified Delphi Method among the department consultants and based on repeated feedback from the users. The final version of the checklist (Figure 1) was used in the care of 502 patients over 13 weeks [12]. For the purpose of the present study, the focus group interviews were conducted after one and five months. During those months, the checklist remained unchanged and was used continuously.

### Participants

To obtain a range of views, the first author (OT, a resident) recruited a purposeful sample of involved nurses, consultant anaesthesiologists, and residents [11,15]. Because views might vary with clinical experience, he recruited the most and least experienced nurses that were on duty during the actual interview day, together with the consultants and residents that were available during, immediately after, or immediately before their shifts. Nine nurses (2-23 years experience; 2 men), four residents (1-4 years experience; 2 men), and one experienced female consultant participated in two focus group interviews. None of the solicited health workers refused to participate.

### Interviews

Each group interview lasted 60 minutes. A resident (OT) and a consultant (GB), both anaesthetists from a different section of our department, moderated and observed the interviews, respectively. To initiate free discussion, the interview guide employed broad, open-ended questions (e.g., "Tell me about your experiences with the checklist use" and "How do you think the checklist use affected daily routines?") [13,14]. Both interviews were transcribed verbatim (by OT). After the first interview, the main findings were identified from the transcript. Based on those findings, we included one additional interview question for the second interview regarding the patients' reactions to the checklist. The discussions in the two interviews were fairly similar; thus, we considered that saturation was achieved after the second interview [14].

### Analysis

Two of the authors (OT and AE) analysed the data using systematic text condensation inspired from Giorgi and modified by Malterud [16,17]. This method comprised four main steps: (1) reading the transcripts to get an overview of the data, (2) identifying text units relevant to our aim and encoding them with codes derived from the data (not determined *a priori*), (3) interpreting similarly coded text units for a common meaning, and (4) summarising the content within the coded groups into descriptions of the participants' views and experiences. Each description was validated by comparing it to the interview context and the data it was based on, by searching the entire transcripts for disproving data, and by member check (the participants validated the findings) [14,16]. The two analysts (OT and AE) contested each other's interpretations of the data and solved disagreements by consensus.

## Results

The participants' views could be summarised with five main statements (Table 1). For each statement, we describe the expressed views in more detail, illustrated with selected quotes (*in italics*).

**Table 1:** Main statement identified in the study

• The checklist could divert attention away from the patient
• The checklist influenced workflow and doctor-nurse cooperation
• Senior consultants were both sceptical and supportive
• The checklist improved confidence in unfamiliar contexts
• The checklist revealed insufficient equipment standardisation

### The checklist could divert attention away from the patient

Participants thought most patients regarded using the checklist as a safeguard or did not notice its use at all. However, there was concern that some patients might become anxious when the checklist was consulted, and that patients who were nervous or needed close attention might feel left alone on the operating table while the health care personnel concentrated on the checklist. One participant speculated that stressed patients might think *"Why are you doing this, are you not properly prepared?" *(Nurse 5).

To reduce these problems, participants reported that they avoided turning their backs to the patient when reading the checklist. Furthermore, when patients needed special attention, the participants conducted much of the checklist before the patient arrived in the OR; *"especially when there is a crying little child waiting in the corridor" *(Physician 4). Some participants said they informed patients of the checklist in advance. One nurse told patients that a checklist would be used in the same way that pilots use them before takeoff. Participants emphasised that they believed that most patients would have a positive experience with the checklist when the focus on the patient was not compromised.

### The checklist influenced workflow and doctor-nurse cooperation

Nurses found that introduction of the checklist interrupted their streamlined pre-induction workflow and caused stress to both the patients and themselves, especially when the physician rushed into the OR and immediately started reading the checklist. The checklist was also said to cause redundant checks by the nurse and the physician. Those problems were reported to occur initially, but diminished over time, as physicians and nurses became accustomed to the checklist and managed to integrate it into their normal working routine. It was also a common experience that the workflow went more smoothly after the involved personnel were able to decide how the checklist should be read and by whom (e.g., two nurses, instead of a physician and a nurse).

The checklist was also perceived to ease doctor-nurse cooperation. One resident said that it felt unpleasant to ask the nurses for additional equipment, but that he no longer had to argue for it after the checklist was implemented. Participants emphasised that *"we must focus on the checklist, not the profession" *(Nurse 1) and that the checking had *"nothing to do with anyone having done a good or bad job" *(Physician 4).

### Senior consultants were both sceptical and supportive

According to some participants, a few experienced consultants were sceptical towards the checklist and might have thought, *" I've done this for thirty years, and now I'm supposed to play around with a checklist" *(Physician 2). One nurse indicated that the checklist was ridiculed and made some consultants in other sections *"laugh a bit" *(Nurse 5). However, several participants emphasised that the head physician's support and motivation were crucial for implementing the checklist. Residents said it would have been very hard to implement the checklist if their chief had had a negative attitude.

### The checklist improved confidence in unfamiliar contexts

Participants valued the equipment control and communication routines achieved in the study section. However, they then felt vulnerable performing anaesthesia elsewhere, where equipment might be *"hidden in some secret place... and nobody helps you" *(Physician 2). To improve confidence in those situations, several participants had used the checklist outside of their department (e.g., in the emergency room, in the coronary catheterisation lab, or when transporting unstable patients). One resident indicated that he always had the checklist in mind outside the OR. It was suggested that the checklist should be adapted to situations other than those for which it was intended.

### The checklist revealed insufficient equipment standardisation

Some participants complained about the differences in both the type and location of equipment among the various ORs: *"The bougie is located in five different places" *(Nurse 3). This eventually resulted in the personnel bringing equipment that was on the checklist into a given OR: "*Instead of searching (for a bougie), we just go and get a new one" *(Nurse 1). As a result, redundant equipment might accumulate in an OR. Participants indicated that using the checklist had highlighted the need for improving equipment standardisation and led to *"things being more in order" *(Nurse 2).

## Discussion

Despite the increasing use of checklists in healthcare worldwide, few studies have explored personnel experiences in using this new tool. Our findings suggest several issues and actions that may be important to consider in implementing and using a checklist (Table 2).

**Table 2:** Issues and actions to consider for checklist use and implementation

• Support and motivation from the head of the department is crucial
• Expect and prepare for sceptical colleagues
• It takes time to become accustomed to checklists; do not draw premature conclusions
• Keep attention focused on the patient during checklist routines
• Inform the patient properly in advance
• Perform part of the checklist before the patient arrives
• Be aware that the checklist may be used in situations for which it was not intended

### The patient must not be forgotten

Study participants' perceptions that the checklist might divert attention away from the patient were unexpected. This issue was not considered prior to implementing the checklist, and we are unaware of any prior study that explored negative patient reactions towards checklists. Negative patient reactions should be considered, validated by speaking with patients, and prevented.

The participants in our study said they reduced negative patient reactions by improving communication with the patient (i.e., facing the patient, informing the patient in advance) and by completing much of the checklist before the patient arrived to the OR. These approaches may be helpful in other situations. Structured and individual information may reduce patient anxiety [18]; in situations that are difficult for the patient, too much focus on a checklist, rather than on the patient, may make the patient's situation worse. We also believe that patient anxiety can be prevented by trained health care workers that are confident about when to read the checklist, and who should lead the session.

### New tools can interrupt workflow

The introduction of the checklist interrupted the established working habits. An aviation-style checklist was a new and unfamiliar tool in our department. In retrospect, it may have been beneficial if we had foreseen this interruption in the existing workflow, because we may have been able to design a plan to prevent it.

Conflicts with the existing organisational structure and culture, such as insufficient communication and lack of time, are well-known barriers to the successful implementation of new guidelines and similar tools [19,20]. The participants in our study solved communication and collaboration problems over time and got used to the checklist. Therefore, conclusions regarding the acceptance of a checklist should not be made prematurely in the implementation process.

### Critical voices must be identified and addressed

Participants reported that the support of the chief physician was the key to success; however, they also reported that experienced consultants made jokes and did not believe in the positive effects of checklists. In a small department, one or a few leading individuals with negative attitudes may cause a stall in new quality-improving projects. It is essential to identify these resistors [21] and allow them to voice their concerns early in the project. Even a mandatory checklist can be difficult, or even impossible, to implement without backing from the organisation's senior leaders [22]. Staff working with quality improvement should not be surprised by scepticism in experienced clinicians concerning the use of checklists. Before considering enforcement measures, sufficient time must be allowed to evaluate the experiences and effects of the checklist and to inform, convince, and accommodate personnel. During the 13-week implementation period, our checklist was not used in 39% of anaesthesias, despite the fact that this was a protocol violation [12]. After this period, the use of the checklist became mandatory and was included in the written quality and safety documentation.

### A checklist may be used in situations for which it was not intended

Standardisation and reliability built into the OR are essential for the safety of anaesthesia [23]. Some participants in our study "exported" the checklist from the OR to remote locations and unfamiliar contexts. Although this showed that the checklist was considered highly useful, "exporting" a checklist to situations in which it was not meant to be used may impede further checklist implementation. To increase the feasibility and usefulness of checklists, WHO emphasises the importance of local adjustment and adaptation [24]. The pre-induction checklist was developed through a step-wise process and was specifically designed for anaesthesia in the contexts of neurosurgery, plastic surgery, burn surgery, and otolaryngological surgery. If the checklist is to be used for other purposes, it should first be revised and validated to ensure that it meets the intended purpose and is safe to use in the new environment. An excessive or improperly designed list could cause checklist fatigue, and could thereby make it difficult to implement a more appropriate list [25].

### Standardisation is important for the usefulness of checklists

Participants observed differences among various ORs in both the type and the location of equipment on the checklist. This lack of standardisation made it more challenging to use the checklist, and this was unforeseen by the project group. After some minor equipment revisions, the ORs became more standardised, and this facilitated consulting the checklist.

### Strengths and limitations of the study

This qualitative study explored more experiences in greater depth than would be possible in a quantitative study (e.g., a survey on pre-selected topics). The strengths of the design included the data transcription by the interviewer, analyses by two researchers, a search for disproving data, and member check (validation of the findings by the participants) [11,14,16]. The study also had limitations. Although focus groups are ideal for exploring common experiences [13], more sensitive, personal issues might have been disclosed in one-on-one interviews. Also, we may have missed some issues because we conducted only two group interviews; however, 41% (14/34) of the checklist users were included in the interviews. Additionally, participants' perceptions of how patients reacted to the checklist should be validated by interviewing patients. The interviewer's interest in checklists was clear to the participants, and this may have made them more willing to report the benefits rather than the difficulties with the checklist; however, it also facilitated the discussion. A range of both benefits and difficulties were actually reported.

## Conclusion

When introducing checklists, the best approach for achieving usefulness and compliance is a complex issue in medicine. Our findings suggest several issues and actions that may be important to consider in checklist use and implementation. Further research should explore checklist acceptability and its effects on the relationships among health professionals.

## Competing interests

The authors declare that they have no competing interests.

## Authors' contributions

OT, GB, and AE designed the study. OT and GB moderated and observed the interviews, respectively. OT and AE analysed the data and drafted the results. All authors made a substantive contribution to writing the discussion and revising the manuscript.

All authors read and approved the final manuscript.

## Pre-publication history

The pre-publication history for this paper can be accessed here:

http://www.biomedcentral.com/1472-6963/10/342/prepub
